# No longer married to inflammasome signaling: the diverse interacting pathways leading to pyroptotic cell death

**DOI:** 10.1042/BCJ20210711

**Published:** 2022-05-24

**Authors:** Ashley Weir, James E. Vince

**Affiliations:** 1 The Walter and Eliza Hall Institute of Medical Research, Parkville, VIC 3052, Australia; 2 The Department of Medical Biology, University of Melbourne, Parkville, VIC 3010, Australia

**Keywords:** apoptosis, gasdermin, GSDMD, inflammasome, necroptosis, pyroptosis

## Abstract

For over 15 years the lytic cell death termed pyroptosis was defined by its dependency on the inflammatory caspase, caspase-1, which, upon pathogen sensing, is activated by innate immune cytoplasmic protein complexes known as inflammasomes. However, this definition of pyroptosis changed when the pore-forming protein gasdermin D (GSDMD) was identified as the caspase-1 (and caspase-11) substrate required to mediate pyroptotic cell death. Consequently, pyroptosis has been redefined as a gasdermin-dependent cell death. Studies now show that, upon liberation of the N-terminal domain, five gasdermin family members, GSDMA, GSDMB, GSDMC, GSDMD and GSDME can all form plasma membrane pores to induce pyroptosis. Here, we review recent research into the diverse stimuli and cell death signaling pathways involved in the activation of gasdermins; death and toll-like receptor triggered caspase-8 activation of GSDMD or GSMDC, apoptotic caspase-3 activation of GSDME, perforin-granzyme A activation of GSDMB, and bacterial protease activation of GSDMA. We highlight findings that have begun to unravel the physiological situations and disease states that result from gasdermin signaling downstream of inflammasome activation, death receptor and mitochondrial apoptosis, and necroptosis. This new era in cell death research therefore holds significant promise in identifying how distinct, yet often networked, pyroptotic cell death pathways might be manipulated for therapeutic benefit to treat a range of malignant conditions associated with inflammation, infection and cancer.

## Introduction

Systemic, tissue and cellular homeostasis is threatened by infection and injury. Surveillance of pathogenic and sterile insults is upheld in multicellular organisms by the innate immune system. The evolutionarily conserved pattern recognition receptors (PRRs) respond to pathogenic virulence factors, environmental toxins and host-derived danger signals to initiate cytokine and chemokine production. In more recent years, the identification of new cell death effectors has highlighted the crucial role of PRRs in signaling cell death during infections, and has uncovered how cell death can act to eliminate intracellular pathogens [1–9]. Conversely, in some contexts, PRR-driven cell death can also increase pathogen infection by limiting the immune response [10–13], or can cause host-tissue damage and increase disease severity, as evidenced in cytokine shock syndromes [14–16].

Inflammasome forming PRRs represent one of the crucial cellular danger surveillance mechanisms that cause both potent inflammatory responses and cell death. These innate immune multimeric protein complexes maintain cytoplasmic homeostasis through the regulation of caspase-1 activation, proinflammatory cytokine maturation, specifically interleukin-1β (IL-1β) and IL-18, and gasdermin D (GSDMD)-driven pyroptosis, a cell death modality that, similar to necroptotic cell death, results in the release of immunogenic molecules [17]. The importance of correct inflammasome functioning in mammalian health has been documented in a multitude of studies detailing how diverse pathogens can overcome host defences when the inflammasome machinery is removed while, conversely, autoactivating mutations and excess inflammasome signaling have been shown to cause both rare hereditary and common autoinflammatory conditions, such as atherosclerosis [1,18,19].

Inflammasome signaling has long been synonymous with pyroptosis. The act of pyroptosis was first described in 1992 as a lytic form of apoptosis occurring in macrophages in response to *Shigella flexneri*-infection [20]. Cookson and Brennan later termed the caspase-1-dependent lytic cell death that they and others observed in *Salmonella* or *Shigella* infected macrophages ‘pyroptosis', derived from the Greek ‘pyro' meaning fire and ‘ptosis' (of piptein), meaning to fall [21–24]. Recently pyroptosis has been redefined as a gasdermin-dependent cell death, following two seminal studies showing that activated caspase-1 (and caspase-11) cleaves the pore-forming protein GSDMD to bring about the cells demise [25,26]. However, with this discovery and the classification of pyroptosis being a gasdermin-driven cell death [27,28], pyroptotic killing is no longer limited to inflammasome signaling. GSDMD and other gasdermin family members incorporating GSDMA, GSDMB, GSDMC and GSDME, can be processed independent of inflammasome complexes, by both cell death associated caspases and other proteases, such as granzymes, to form plasma membrane pores and trigger pyroptotic cell death [29]. These gasdermins are generally composed of a N-terminal pore-forming domain, though the gasdermin family member Pejvakin (PJVK) is an exception to this, an interdomain linker that is cleaved to unleash gasdermin activity, and an autoinhibitory C-terminal domain. The pore-forming ability of the N-terminal domain of gasdermin proteins is highly conserved, with mammalian pore-forming family members (GSDMA-E) having arisen from genetic duplications of a gasdermin gene present in ancient metazoans, suggesting a high degree of evolutionary pressure [30,31]. Experimental studies also show that the N-terminal domain of coral and lancelet gasdermin are both capable of causing pyroptosis, indicating that gasdermin function is conserved beyond mammals [31,32].

Unlike pyroptosis, necrotic cell death represents a passive cell lysis caused by overwhelming chemical, metabolic or physical stress that is not genetically encoded. While both types of cell death are viewed as highly inflammatory, due to membrane rupturing that allows the release of immunogenic content, gasdermin pore formation results from cellular sensing of specific host or pathogen-derived molecules and highly regulated cell signaling pathways, and as such, its activity can be finely tuned. The regulation of gasdermin pore formation, in addition to its size and charge preference for specific molecules such as IL-1β, can control the generation and release of specific inflammatory cytokines. Indeed, quantitative proteomics of cells undergoing pyroptosis has highlighted GSDMD-mediated release of smaller sized proteins, including IL-1β as well as alarmins (e.g. Galectin 3 and HMGB1) and luminal lysosomal proteins, even when cell lysis is blocked [33]. As discussed below, the modulation of the gasdermin pore can therefore act to delay, or even abrogate, pyroptotic cell death in some cell types such as neutrophils, thereby ensuring a prolonged anti-pathogen inflammatory response.

In mammals, recent insights into the molecular signals and proteases that trigger gasdermin cleavage to cause pyroptosis, and the networking of other cell death modalities with pyroptosis, including apoptosis, necroptosis and even ferroptosis-associated events, has arguably heralded in a new era of programmed cell death research. In this review we discuss the growing number of studies supporting the interplay between inflammasome activation, pyroptosis and its role in alternate cell death signaling pathways. These findings highlight how distinct programmed cell deaths can contribute to inflammasome activation to cause inflammation, how inflammasome signaling can bypass GSDMD and activate apoptosis, and conversely, how the apoptotic cell death machinery can act in a lytic manner by activating specific gasdermin family members to cause pyroptosis. We suggest that this emerging intricate web of cell death signaling underlines the importance of cell death for blocking microbial replication and exposing pathogens to immune-mediated attack, and that the repurposing of targeted anti-cancer cell death therapeutics represent a promising new class of anti-pathogen and immunomodulatory treatments.

## A conventional look at pyroptosis: canonical and non-canonical inflammasome-triggering of GSDMD

**Figure 1.**
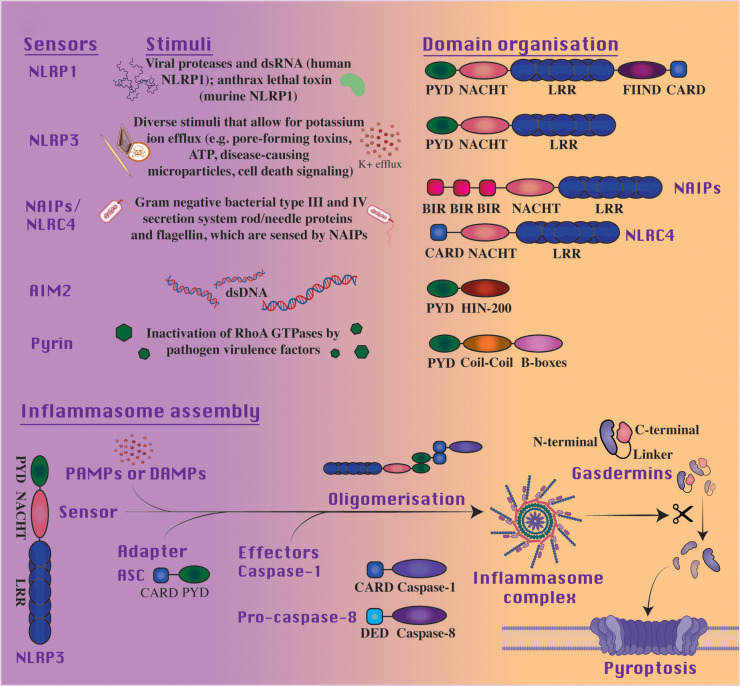
Inflammasomes share structural similarities despite being stimulated by different signals. Inflammasome sensors are activated by differing stimuli though share similarities in domain organization. Within the NLR family, receptors are generally characterized by a tripartite structure, with an N-terminal signaling domain, a central nucleotide-binding domain (NBD) and a leucine-rich repeat (LRR)-containing C-terminal domain, involved in ligand binding or agonist sensing. Members of the NLR family can be partitioned into NLRPs or NLRCs through the variability of the N-terminal regions, typically containing a pyrin or a caspase activation and recruitment domain (PYD or CARD, respectively), in support of protein–protein interactions. Of these receptors, NLRP1, NLRP3 and NLRC4 are recognized inflammasome sensors, although NLRC4 assembly can occur with or without the ASC adaptor. Alternatively, the ALR family is defined structurally by an N-terminal PYD domain and a DNA binding C-terminal hematopoietic interferon-inducible nuclear protein with 200-amino acid repeat (HIN-200) domain. Of the ALR family, Absent in melanoma 2 (AIM2) is the best characterized. The N-terminal of the pyrin inflammasome contains a PYD to support homotypic interaction with the ASC adaptor and subsequent caspase recruitment, while a Coil–Coil domain can be identified in a central region with a B-boxes domain at the C-terminal. Broadly, with signaling by PAMPs or DAMPs these inflammasome sensors, for example NLRP3, oligomerize with effectors through adapter proteins to form inflammasome complexes. Subsequently, activated effector protein cleavage of gasdermins at their linker region releases the pore-forming N-terminal to bring about pyroptotic cell death.

Three canonical inflammasome components exist that are required for the formation of an active inflammasome complex; an inflammasome sensor protein, an adaptor, and zymogenic caspase-1. Activation is initiated through inflammasome forming PRRs in response to cytoplasmic pathogen-associated molecular patterns (PAMPs), host-derived damage-associated molecular patterns (DAMPs) or environmental dangers [34]. Intracellular sensors of the prior stimuli are distinguished by structural features described in Figure 1; they include members of the nucleotide-binding and oligomerization domain (NOD)-like receptor (NLR) and absent in melanoma (AIM2)-like (ALR) receptor families and pyrin. While NLRP3 has been the most frequently investigated PRR in canonical inflammasome triggering due to its sensing of diverse cellular threats, NLRP1, NLRC4, AIM2 and pyrin are all capable of similar pathway activation. Further, autoactivating mutations in the inflammasome forming NLRs and pyrin cause autoinflammatory syndromes [18]. In all instances the inflammasome sensors oligomerize following PAMP or DAMP detection, which allows recruitment of the adaptor molecule apoptosis-associated speck-like protein containing a caspase activation and recruitment domain (ASC). The bipartite structure of ASC, containing a pyrin domain (PYD) and a caspase recruitment domain (CARD), facilitates homotypic interactions between both PYD containing receptors and the CARD containing caspase-1. Caspase-1 recruitment, and its dimerization, results in proximity-induced autocatalytic cleavage and activation. Distinctly, function-to-find domain (FIIND) cleavage and NLRP1's C-terminal CARD (which is inhibited by the protease DPP9), and not the PYD, allow for ASC and caspase-1 recruitment [35–38]. In addition, unlike PYD-containing NLRs, NLRC4 can recruit caspase-1 directly via its CARD to facilitate cleavage and subsequent cytokine maturation [39].

Active caspase-1 mediates the cleavage of IL-1β and IL-18, in addition to the pyroptotic effector, GSDMD. A central linker region separating the pore-forming N- and autoinhibitory C-terminals of GSDMD is cleaved at the site _272_FLTD|G_276_ in human GSDMD (hGSDMD) and _273_LLSD|G_277_ in murine GSDMD (mGSDMD) by caspase-1 (and caspase-11 in mouse or caspase-4 and -5 in human) to cause its activation [25,26]. This cleavage allows cardiolipin, phosphatidylinositol 4-phosphate, phosphatidylinositol 4,5-biphosphate (PIP2), and/or phosphatidylserine membrane lipid binding by the N-terminal that induces conformational changes and oligomerization, resulting in the formation of 215 Å GSDMD pores in the plasma membrane, non-selective ionic fluxes and cell death [25,26,40–43]. The preferential redistribution of mature IL-1β and IL-18 to PIP2 lipids within the plasma membrane is dictated by their net positive charge imparted by a polybasic motif, which may become exposed upon caspase-1 cleavage, and the subsequent exit of IL-1β and IL-18 from the cell is preferentially engaged by a negatively charged GSDMD conduit [42,44]. Furthermore, the decoration of precursor IL-1β by ubiquitin chains acts to target it for degradation and prevent its processing by caspase-1 [45]. Recent reports indicate that bacterial effectors or viral proteases can also target GSDMD for ubiquitination and degradation, or inactivating proteolysis, respectively, to block host cell death and enable efficient infection [46–49], while other viral proteases may activate GSDMD killing activity [50,51]. The loss of GSDMD can protect mice from IL-1-driven inflammation caused by autoactivating NLRP3 inflammasome mutations, highlighting the importance of the GSDMD pore in not just eliciting cell death, but also allowing for efficient IL-1β release [52,53]. However, in other IL-1β-driven inflammatory models, such as K/BxN serum transfer-induced arthritis and gout-associated uric acid crystal-induced peritonitis, GSDMD does not drive disease severity, which may reflect inflammasome-independent IL-1β activation and cell death [54,55].

**Figure 2.**
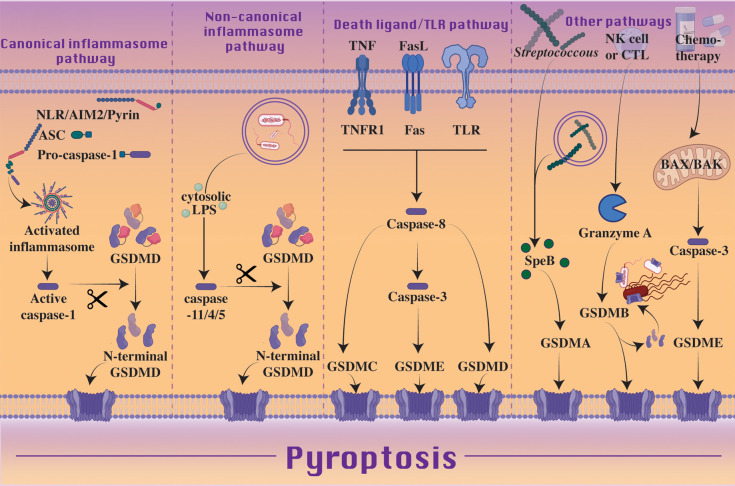
Pyroptosis is executed by the gasdermin pore-forming family of proteins. Canonical inflammasome signaling can activate pyroptosis through sensing of potentially injurious agents, including bacteria, toxins, viruses, ATP, crystalline material, and DAMPs. Inflammasome sensors, NLRs, AIM2 or pyrin, assemble into large multimeric protein complexes alongside adaptor proteins such as ASC and effectors such as caspase-1. Activation of the inflammasome results in caspase-1 cleavage of GSDMD, releasing the pore-forming N-terminal effector of pyroptosis. IL-1β and IL-18 are also processed by caspase-1, and released through GSDMD pores. Caspase-11/4/5 activation downstream of cytosolic lipopolysaccharide (LPS) represents the noncanonical pathway to GSDMD cleavage and pyroptosis, though in this instance, cytokines are not processed. Alternatively, ligation of death receptors or TLRs by their cognate ligands, can signal caspase-8 activation. Caspase-8 can then trigger GSDMC/D/E pore formation, though a caspase-3 intermediate step is required for GSDME processing. Chemotherapy-induced pyroptosis has been reported to occur in a similar manner, with BAX and BAK activation followed by caspase-3/7 resulting in GSDME pore formation. Streptococcus-associated protein SpeB and Granzyme A of natural killer cells and cytotoxic T lymphocytes have been shown to trigger GSDMA and GSDMB, respectively, to initiate pyroptosis. The N-terminal of GSDMB also has the propensity to directly target and lyse bacteria, including *Shigella*, *E. coli*, *Citrobacter* and *Salmonella*.

**Figure 3.**
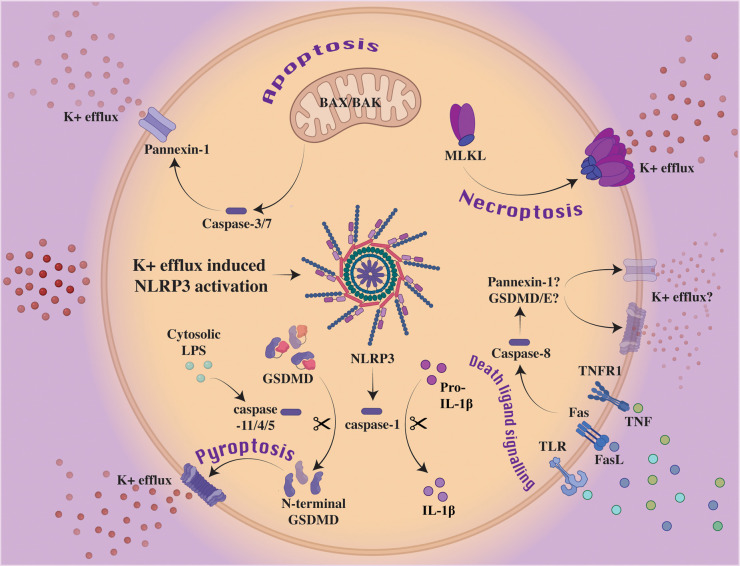
The NLRP3 inflammasome is activated downstream of several cell death signaling pathways via potassium ion efflux. Classically, NLRP3 processes caspase-1 to support its subsequent cleavage of GSDMD and the cytokines IL-1β and IL-18. Potassium ion efflux is a common determinant required to activate NLRP3 in nearly all circumstances. Non-canonical inflammasome triggering allows for potassium ion efflux via GSDMD pores, which causes NLRP3 oligomerization and signaling. Similarly, downstream of necroptotic MLKL oligomerization and membrane permeabilization, potassium ion efflux has also been reported to activate NLRP3. Proapoptotic proteins, BAX and BAK, are known to trigger similar potassium ion efflux in a caspase-3/7 and pannexin-1 channel dependent manner. The potential role of pannexin-1 and/or GSDMD/E in activating NLRP3 downstream of death ligand signaling to capase-8 remains a question of interest.

Non-canonical inflammasome signaling results upon exposure of gram-negative bacterial lipopolysaccharide (LPS) to cytosolic caspase-11 (caspase-4 and -5 in humans) [25,56,57] (Figure 2). Guanylate-binding proteins (GBPs) are not only proposed to mediate the lysis of vacuole membranes containing bacteria [58,59] and facilitate the disruption of cytosolic pathogen membranes [60,61], but have been shown to present LPS to caspase-11/4/5 by behaving as polyvalent signaling platforms [62]. Specifically, GBP1 appears to initiate platform assembly through electrostatic association with LPS or cytosolic *Salmonella*, with GBP2 and 4 being suggested as mediators, while GBP3 likely governs caspase activation [61,63–65]. Through GBP-mediated exposure of LPS, caspase-11 interacts with the LPS lipid A moiety resulting in caspase-11 activation and processing of GSDMD into the pore-forming N-terminal fragment although, unlike caspase-1, caspase-11 does not directly process IL-1β [66–72]. Instead, the GSDMD pore allows for cellular potassium ion efflux, which is a common determinant for NLRP3 inflammasome triggering in response to diverse stimuli and cellular stressors [73–76] (Figure 3). Thereby, the non-canonical caspase-11-GSDMD inflammasome can indirectly induce NLRP3 signaling and IL-1β and IL-18-driven inflammatory responses. This same triggering of NLRP3 via the GSDMD pore and potassium ion efflux has also recently been proposed to occur downstream of a TLR or death receptor-caspase-8-GSDMD axis [77,78] (Figure 3). Contrary to canonical inflammasome signaling, death through non-canonical signaling has not been observed in the absence of GSDMD, though as caspase-7 is a recently proposed substrate of caspase-4, further investigation into cross-talk with apoptotic pathways is warranted [79].

## GSDMD pore function: more than just cell death

A number of studies have now identified diverse cellular functions for the GSDMD pore. In neutrophils, non-canonical inflammasome activity and GSDMD targeting of the nuclear membrane results in neutrophil extracellular trap formation to control *Salmonella* infection [80], while in macrophages it has been reported that both non-canonical and canonical inflammasome signaling and GSDMD pore formation causes calcium ion influx to allow for IL-1α maturation [81]. GSDMD activation and potassium ion efflux downstream of dsDNA-AIM2 inflammasome activation can also act to inhibit dsDNA-induced cGAS/STING signaling, thereby limiting type I interferon (IFN) production [82,83]. This function of GSDMD was reported to occur independent of pyroptosis, and the specific increase in IFNβ production, not bacterial numbers, in AIM2 deficient animals increased their susceptibility to lethal *Francisella novicida* infection. Mechanistically, it was shown that GSDMD loss and maintenance of intracellular potassium ion levels allowed increased cGAS oligomerization and enzymatic activity, and this was a direct result of potassium ions promoting cGAS catalytic activity. In contrast, GSDMD mitochondrial membrane targeting following non-canonical inflammasome activation has been reported to release mitochondrial DNA (mtDNA) into the cytoplasm, to activate cGAS and suppress endothelial cell proliferation, and hence regeneration, following lung injury [84]. Interestingly, in addition to nuclear GSDMD targeting in neutrophils, the N-terminal of GSDMD has also been reported to preferentially accumulate in primary granules and autophagosomes, as opposed to the plasma membrane [85]. This distinct intracellular targeting of neutrophil GSDMD may therefore limit neutrophil pyroptosis and can induce autophagy-dependent IL-1β secretion [85,86]. The release of granule stored neutrophil elastase into the cytosol can also act on GSDMD to generate a N-terminal pore-forming fragment which, in this case, was associated with neutrophil death and, therefore, the loss of GSDMD increased neutrophil levels and the hosts ability to combat extracellular *E. coli* infection [13]. More recently, intestinal epithelial cell GSDMD was shown to mediate goblet cell mucin secretion independent of cell death, and as such GSDMD was important in the formation of the mucus layer [87]. Therefore, GSDMD pores may serve several purposes beyond cell death and IL-1β release.

## Death receptor and caspase-8 activation of GSDMD to drive pyroptosis

**Figure 4.**
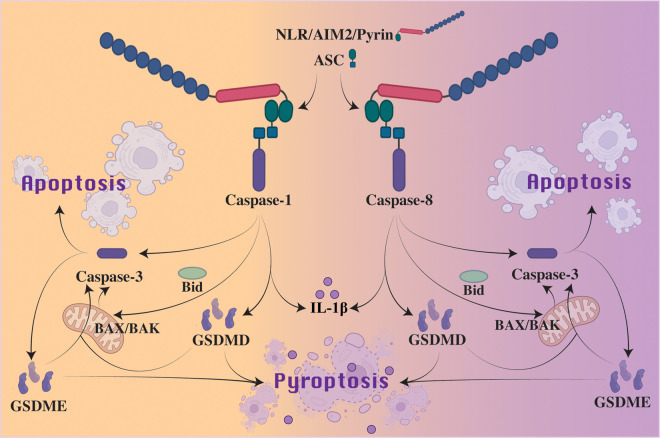
Cross-talk between apoptosis and pyroptosis following inflammasome activation. Effectors recruited to the inflammasome complex, such as caspase-1 and caspase-8, prompt strikingly similar downstream signaling. Cross-talk between caspase-3 and caspase-1 or -8 following inflammasome activation can induce apoptosis. Through inflammasome-mediated caspase-1 or caspase-8 cleavage of Bid, some studies have suggested that inflammasomes can initiate BAK and BAX signaling to activate caspase-3 to bring about apoptosis. Caspase-8 has also been shown to directly cleave GSDMD, thus curtailing apoptosis to induce pyroptosis. Furthermore, when activated by caspase-1 or caspase-8, caspase-3 can cleave GSDME to release N-terminal pores and initiate pyroptosis. Activated GSDMD and GSDME may also directly target mitochondrial membranes to induce a non-canonical mitochondrial apoptosis that results in apoptosome formation, and hence initiate a feedback caspase-3 amplification loop. It should be noted that in commonly studied inflammasome-responsive cell types, such as macrophages, the primary cell death observed from inflammasome sensor engagement is caspase-1-driven GSDMD activation and pyroptosis. However, alternate cell death pathways are often engaged upon (i) prolonged inflammasome signaling, (ii) reduced levels of activating stimuli (e.g. a lower level of cytosolic DNA has been shown to engage AIM2-mediated caspase-8 activation and apoptosis), or (iii) altered levels of relevant cell death effectors, such as loss, or reduced expression, of caspase-1 or GSDMD.

Despite being classified as an apoptotic initiator caspase, caspase-8 has been described to function similarly to caspase-1 [88]. Following TLR or death receptor triggering, or fungal-derived β-glucan activation of dectin-1, activated caspase-8 can cleave precursor IL-1β into its bioactive fragment at the same site as caspase-1 [89–94]. Similarly, caspase-1 has a reported capacity to cleave Bid into its pro-apoptotic fragment, tBid, to drive BAX and BAK activation and mitochondrial apoptosis, a function typically assigned to caspase-8 [95–97] (Figure 4). Finally, recent studies have shown that caspase-8, like caspase-1 and -11, can also directly cleave GSDMD into its N-terminal pore-forming fragment to trigger pyroptosis; upon TNF-induced lethality, deletion of the caspase-8 inhibitor cFLIP or following *Yersinia* infection and/or the inhibition of NF-κB signaling [78,98–100] (Figure 2). On the other hand, catalytically inactive caspase-8 generated through gene editing, which cannot process GSDMD, appears to act in a scaffolding capacity to engage ASC and caspase-1 to cause spontaneous lethality in mice, and this even occurs on an MLKL deficient background (to prevent the necroptotic activity that is unleased by a loss of caspase-8 catalytic function) [101,102]. While GSDMD deficiency did not confer a survival advantage upon loss of caspase-8 catalytic activity, this may be a result from catalytically inactive caspase-8 still being able to drive caspase-1-mediated apoptotic caspase-3 and -7 activation.

## Apoptotic activation of pyroptosis by caspase-3 cleavage of GSDME

Both death receptor and intrinsic mitochondrial (BAX and BAK-dependent) apoptosis converge on the activation of the apoptotic effector caspases, caspase-3, -6, and -7. While these caspases can dismantle the cell to limit its immunogenic potential, caspase-3 has recently been documented to cleave (at _267_ DMPD _270_) and activate GSDME (or DFNA5, deafness autosomal dominant 5), a gene implicated in hearing loss and often silenced in cancer [103,104]. Consequently, caspase-3 triggering of GSDME can cause pyroptosis downstream of viral, chemotherapy-induced mitochondrial, or death receptor-mediated, apoptotic signaling (Figure 2) [103,105]. In mammals, the expression levels of GSDME likely dictate whether this pyroptosis follows apoptotic signaling events (historically referred to as secondary necrosis), or is preferentially engaged upon caspase-3 activation [103,105]. Therefore, it will be important that future studies examining the physiological consequences of caspase-3 and -7-mediated cell death also examine the potential role of GSDME in generating the relevant phenotype, either as the primary driver of cell death, or through initiating the secondary necrosis that can succeed apoptosis.

The pyroptotic function of GSDME is evolutionarily conserved in coral, where coral infection with *Vibrio coralliilyticus* can trigger caspase-3 and GSDME to cause cell death [32]. A role for GSDME-induced pyroptosis and bactericidal activity in the marine teleost *Cynoglossus semilaevis* has also been proposed through its cleavage by caspase-1 and to a lesser extent caspase-3/7, with preservation of caspase-1 cleavage of GSDME being observed in many teleost species [106]. Furthermore, while GSDMA-GSDMD orthologs appear restricted to mammals, phylogenetic analysis of GSDME in invertebrates, including hydra, molluscs, brachiopods, corals, and sea anemones indicates a closer relation to mammalian DFNB59 (or PJVK) which, unlike other mammalian gasdermins, does not cause pyroptosis when the N-terminal fragment is expressed in cell lines [31]. Recent work reported a gasdermin N-terminal homolog, regulator of cell death 1 (RCD1), in the filamentous fungi *Neurospora crassa*, which like gasdermins localizes to peripheral cell margins, interacts with phospholipids including cardiolipin and when expressed in 293T kidney cells, can induce a pyroptosis-like death [107]. Although these results illustrate similarities in cell death execution proteins between the fungal and animal kingdoms, the level of RCD1 sequence identity with mammalian gasdermins has been disputed, and the lack of *Rcd* genes in organisms beyond fungi and bacteria have led others to suggest that RCD1 should not be considered a gasdmerin family member [31]. Regardless, it appears that, at least functionally, gasdermin-like pore-forming proteins predate the metazoan era.

Although mammalian GSDMD loss prevents non-canonical inflammasome triggered cell death, it limits, but does not abrogate, cell death induced by canonical inflammasomes, including NLRP3, NLRC4, NLRP1 and AIM2. This appears to be a result of the ability of ASC to either (i) recruit caspase-8 and thereby engage caspase-3, largely when caspase-1 or GSDMD activity is lost [108–113], or (ii) trigger caspase-1 activation of caspase-3 [96,113,114] (Figure 4). The activation of caspase-3 by canonical inflammasome activity can, in some circumstances, result in GSDME processing and pyroptotic cell death. For example, in LPS or TNF challenged NLRP3 autoactivating mice, high levels of GSDMD-independent IL-1β and IL-18 release is observed, which largely results from GSDME activity [115]. Alternatively, as observed for the NLRC4 inflammasome, caspase-8 recruitment and activation does not always cause GSDME-driven pyroptosis [109], which may reflect limited GSDME expression levels or the ability of NINJ1 to cause cell lysis in its absence [116].

Interestingly, in caspase-1 deficient cells *Francisella novicida* mediated AIM2-caspase-8 inflammasome-induced cell death was reduced by inhibiting mitochondrial BAX and BAK activation [117]. This finding is similar to that reported in a more recent study showing that inflammasome mediated caspase-1 and caspase-8 triggering of Bid could cause mitochondrial membrane permeabilization, with subsequent caspase-3 activation inducing cell lysis when GSDMD was genetically deleted [97]. While no defect in cell lysis was observed upon GSDME deletion (possibly reflecting NINJ1 activity), the results largely mirror a recently described caspase-1-driven pyroptosis, whereby cleavage of Bid is mediated by caspase-1, with consequent mitochondrial apoptotic triggering and downstream caspase-3 activation of GSDME [96]. Notably, this pyroptotic cell death was implicated in cell types that naturally express low levels of GSDMD, such as cortical neurons and mast cells, and speculatively, may also take place upon pathogen-mediated inhibition of GSDMD [46,47,49]. While the induction of mitochondrial apoptosis resulting from caspase-8 or caspase-1 signaling result, at least in part, from caspase-8 or caspase-1 cleavage of Bid to activate BAX and BAK, it is worth noting that caspase-8 can also promote BAX and BAK activity independent of Bid, by regulating the transcription of Bcl-2 family members [14]. Whether caspase-1 may also control gene transcription [118,119], akin to caspase-8 processing of N4BP1 to control TLR-induced transcriptional activity [120], will be of interest to determine.

Despite the above findings, the consequences of GSDME deletion to chemotherapy responses and cellular homeostasis in infection*,* remain to be fully explored. However, recent research highlights a potential role for caspase-3 and GSDME in causing pyroptosis in eye diseases-associated with all *trans*-retinal-mediated photoreceptor degeneration [121]. Emerging studies also indicate that GSDME expression in cancer cells can in some [105], but not all [122], situations enhance anti-tumor immune responses to limit tumor growth, or can drive neutrophil IL-1β release and pyroptosis downstream of RIPK1 and caspase-3 activation to protect the host from infection by the gram negative bacterial pathogen *Yersinia* [123]. On the other hand, GSDME-induced pyroptosis and the consequent inflammatory response contributes to H7N9 influenza-mediated lethality in mice [124]. Recent findings also correlate GSDME levels and activation in patient intestinal mucosa or synovial immune cells with Crohn's disease and rheumatoid arthritis severity, respectively, and mice deficient in GSDME were protected from chemical-induced (2,4,6-trinitrobenzenesulfonic acid) colitis or collagen-induced arthritis [125,126]. These studies highlight how an excess of GSDME-driven cell death can cause pathological inflammatory responses.

## Death receptor induced pyroptosis via activation of GSDMC

Recent evidence has linked the death receptor initiator caspase, caspase-8, to the cleavage of GSDMC and pyroptosis (Figure 2). Under hypoxic conditions the immune checkpoint inhibitor PD-L1 (programmed death ligand 1) was reported to interact with phosphorylated STAT3 in cancer cells to induce the transcription of GSDMC [127]. Consequently, upon TNF-induced caspase-8 activation, caspase-8 cleaved GSDMC at _362_ LELD _365_ to release a pore-forming N-terminal domain causing cancer cell pyroptosis. Although cleaved by caspase-8, GSDMC was not cleaved by the highly homologous death receptor initiator caspase, caspase-10, which, while not present in mice, in humans can act like caspase-8 to cleave caspase-3 and -7 downstream of death receptor activation. Tumor associated macrophages were identified as a source of TNF *in vivo* able to trigger GSDMC-driven cancer cell pyroptosis although, unlike other studies implicating pyroptosis as triggering cancer cell immunogenicity [105,128], increased GSDMC-expression has been linked to promoting tumor growth and/or inhibiting anti-tumor immune responses [127,129]. The metabolite α-ketoglutarate was also suggested to cause caspase-8-mediated GSDMC processing and pyroptosis in cancer cells via death receptor-6 signaling, although in this case GSDMC expression was beneficial in decreasing tumor size and metastasis (following α-ketoglutarate treatment) in murine xenograft cancer models [130]. Whether hypoxia or α-ketoglutarate can induce innate immune cell GSDMC expression to alter death receptor signaling responses outside of the cancer setting, or how GSDMC deficient mice (four murine GSDMC orthologues exist) respond to genetically and/or chemically induced cancer development, or infection, remains to be full explored. However, the gastrointestinal roundworm *Nippostrongylus brasiliensis*, the tricarboxyclic acid cycle (TCA) intermediate succinate, and the type 2 cytokines, IL-4 and IL-13, can increase GSDMC expression in mouse intestinal organoids, and pore-forming GSDMC is detected in the intestine of mice infected with *N. brasiliensis*, suggesting GSDMC-induced pyroptosis may regulate intestinal cell death resulting from type 2 immune responses [131].

## Granzyme A processing of GSDMB to cause pyroptosis

Although GSDMB has no orthologs in mice, which makes the analysis of its physiological function more difficult, GSDMB was initially shown to cause pyroptosis when its N-terminal domain was expressed in 293T cells [43]. More recently it was demonstrated that natural killer cells and cytotoxic T lymphocytes release Granzyme A into target cells via perforin, resulting in Granzyme A cleavage of GSDMB at K244 (and K229) into its pore-forming fragment to cause target cell pyroptosis [132] (Figure 2). Interestingly, IFNγ, type I IFNs and to a lesser extent TNF, could induce GSDMB expression in some cancer cell lines, and IFNγ treatment could thereby confer susceptibility to Granzyme A-driven pyroptosis *in vitro*, while the expression of GSDMB in tumor cells facilitated tumor clearance *in vivo* following immune checkpoint blockade [132]*.* The delivery of Granzyme A into host cells containing bacteria was also shown to activate GSDMB, but in this case GSDMB was suggested to directly target bacterial membranes enriched in the lipid cardiolipin (e.g. *Shigella, E. coli, Citrobacter* and *Salmonella*) to cause bacterial lysis independent of host cell death, and that pore-forming GSDMB was comparatively inefficient at binding mammalian liposomes [133]. The *Shigella* effector, IpaH7.8 was also shown to target GSDMB for proteasomal degradation [133], akin to its reported targeting of human (but not mouse) GSDMD [46], indicating that pathogens may target multiple gasdermin family members at the same time to shut down a cells pyroptotic capacity. It has been suggested that IpaH7.8 targets GSDMB but not GSDMD for degradation [133], though this conclusion may be attributed to findings demonstrating that epitope tagging of the N-terminal of GSDMD disrupts degradation by IpaH7.8 [46]. However, other incongruities in the GSDMB literature remain to be resolved, including reports that the N-terminal of GSDMB does not bind cardiolipin, but preferentially binds phosphoinositides or sulfatide [134], and that GSDMB does not directly cause pyroptosis, but associates with caspase-4 to promote its activity and non-canonical inflammasome mediated cell death [135].

GSDMB polymorphisms and/or expression levels have been associated with allergic and inflammatory diseases, such as asthma and inflammatory bowel disease (IBD) [134–141], with some disease-associated GSDMB variants linked to decreased activity [142,143]. Mice engineered to express human GSDMB develop an asthma phenotype [139], while in IBD increased GSDMB expression in colonocytes was reported to promote intestinal epithelial repair independent of its pyroptotic signaling capacity [143]. GSDMB expression can also correlate with a positive or negative prognosis depending on the cancer type [132,144]. Whether GSDMB cell death-independent activity might contribute to GSDMB-associated cancer cell growth in some contexts [145,146] remains to be fully investigated, although it has been speculated that GSDMB binding to signaling lipids may impart non-pyroptotic functions [134].

## Bacterial activation of GSDMA to trigger pyroptotic death

The N-terminal of GSDMA3 (three homologs of GSDMA exist in mice) forms pores with a diameter of ∼180 Å and, similar to GSDMD, GSDMA binds phosphoinositides and cardiolipin [40,43,147]. Recent research has uncovered how GSDMA, which is highly expressed in the skin epithelia, is activated by group A *Streptococcus* (GAS) via the bacterial protease SpeB, which processes GSDMA after Q246 in the linker region to unleash the N-terminal pore-forming fragment [148] (Figure 2). Mice lacking GSDMA1 showed increased systemic infection and mortality upon infection with GAS, highlighting the important role of GSDMA in sensing pathogen virulence factors to protect the host. Whether GSDMA is cleaved by other microbial or host cell proteases, and if GSDMA variants linked to autoimmune diseases with skin pathologies, such as systemic sclerosis [149], alter GSDMA activity, will be of significant interest to determine.

## Tuning pyroptotic activity and cell lysis via host cell factors: apoptotic caspases, free radicals, membrane shedding and NINJ1

The intricate control of inflammasome signaling and other cell death modalities is required to prevent pathogen infections and inflammatory diseases [150,151]. As such, it comes as no surprise that pyroptotic cell death and cell lysis are tightly controlled at the genetic level, and can be evaded or even overridden, thus highlighting the essential role of its regulation in responding to cellular threats while attempting to limit collateral damage to the host.

Reactive oxygen species (ROS) have long been implicated in activating the NLRP3 inflammasome, although its exact roles are still debated [152]. More recently, the loss of glutathione peroxidase 4 (GPX4), which acts to prevent free radical generation and lipid peroxidation, was shown to promote caspase-11 and GSDMD-driven pyroptosis in a murine sepsis model. Lethality was reduced by administration of the antioxidant vitamin E or the deletion of caspase-11 and GSDMD but, perhaps surprisingly, not by the inhibition of ferroptotic cell death (a caspase-independent cell death often triggered by GPX4 removal and overwhelming lipid peroxidation) [153]. GPX4 deficiency increased both canonical and non-canonical inflammasome activation of caspase-1 and caspase-11, respectively, to enhance GSDMD processing. Notably, pyroptosis caused by expression of the pore-forming N-terminal domain of GSDMD alone was also exacerbated in GPX4 deficient macrophages, and this effect was blocked by vitamin E supplementation, or the targeting of phospholipase C (PLC) and its ability to initiate calcium signaling via hydrolysis of PIP2. These findings support the notion that increased free radical production is a significant driver of GSDMD-mediated pyroptosis, and that GSDMD activity at the plasma membrane may be facilitated by phospholipid peroxidation and calcium mobilization.

Mitochondrial ROS have also been implicated in pyroptosis. It has been suggested that mitochondrial ROS oxidize four cysteine residues of GSDMD (Cys38, 56, 268 and 367) to facilitate caspase-1 cleavage of GSDMD [154], while recent work indicates that mitochondrial ROS also promote N-terminal GSDMD oligomerization at the plasma membrane [155]. In the later study, a genetic screen was conducted to identify factors that are required to prevent pyroptotic cell death upon inducible expression of the pore-forming N-terminal fragment of GSDMD. This identified the Ragulator-Rag complex, previously implicated in metabolic sensing as well as lysosome and mitochondrial homeostasis, as stimulating GSDMD-mediated killing. The deletion of the Ragulator-Rag complex components, the GTPases RagA, RagB or RagC, and Raptor, a component of the mTORC1 complex that is activated by Ragulator-Rag signaling, reduced N-terminal GSDMD-mediated cell death upon its expression, or NLRC4 inflammasome activation and pyroptosis, by ∼50%. Notably, GSDMD oligomerization, but not GSDMD cleavage nor membrane localization, was reduced upon the loss of Ragulator-Rag signaling, and this was shown to result from a protection from N-terminal GSDMD-mediated decreases in mitochondrial membrane potential and increased ROS generation. Therefore, these findings suggest that cleaved GSDMD activates the Ragulator-Rag complex and mTORC1 to induce mitochondrial ROS, which subsequently facilitates plasma membrane GSDMD oligomerization and pore formation. Intriguingly, and in contrast with these findings, in a screen for genes involved in either caspase-8- or caspase-11-dependent cell death, it was reported that the Ragulator-Rag complex was not required for either canonical or non-canonical driven pyroptosis, but was required for caspase-8-mediated macrophage killing via its assembly of the death-inducing RIPK1 and caspase-8 complex on lysosomal membranes [156]. The reasons for these opposing findings will be important to evaluate. Regardless, a significantly altered cellular metabolism resulting from GSDMD activity is consistent with a single cell analysis indicating that GSDMD may initiative several cellular events, such as ion channel opening, calcium influx, and mitochondrial depolarization, prior to a loss of plasma membrane integrity [157]. Whether other gasdermins may impart similar changes to cellular homeostasis, and are also regulated by free radical production, remains to be tested.

Rupture of the plasma membrane upon gasdermin pore formation was considered a passive process until recent work by Kayagaki et al. [116] demonstrated that a conserved extracellular α-helix of NINJ1 was a mediator of cell lysis downstream of pyroptosis- and apoptosis-induced cell death. Although NINJ1-deficient bone marrow derived macrophages display attenuated LDH release (a measure of cell lysis), NINJ1 was dispensable in the formation of GSDMD pores, cell death and the release of IL-1β and IL-18 via canonical and non-canonical inflammasome signaling. Notably, NINJ1 deficient mice were susceptible to *Citrobacter*-mediated lethality, thereby implicating this cell lysis in protection from bacterial infection. Similarly, Bjanes et al. [158] noted significantly reduced NINJ1 expression protected macrophages from lytic death, including forms induced by canonical and non-canonical inflammasome signaling. NINJ1 has also been documented to mediate membrane rupture and efficient LDH release following a caspase-8 and inducible nitric oxide synthase (iNOS)-mediated cell death caused by IFNγ and pathogen-ligand TLR signaling [14]. It is tempting to speculate that NINJ1 may therefore contribute to the damaging caspase-8- and iNOS-driven host response observed following SARS-CoV-2 infection [14], however how NINJ1 causes cell lysis requires further study.

Recently, Santa Cruz Garcia and colleagues provided evidence that GSDMD pores are capable of both opening and closing in a phosphoinositide-dependent manner to mediate calcium influx, potassium ion efflux and, with sustained pore opening, bring about cell death [159]. Interestingly, as highlighted by the impact of GPX4 deficiency on lipid peroxidation, PLC activity and GSDMD pore formation, these results further suggest the potential for lipid membrane dynamics to play a regulatory role in pyroptosis, as opposed to merely offering membrane support to GSDMD pores, and should stimulate further research. Phosphoinositides are also suggested recruiters of the endosomal sorting complex required for transport (ESCRT) machinery for membrane repair, which has been identified as a negative regulator of necroptosis by facilitating the shedding of MLKL damaged membranes [160]. Similarly, Rühl et al. [161] observed that upon LPS delivery into the cytosol of macrophages, calcium influx occurred in a GSDMD and caspase-11 dependent manner, which was able to recruit the ESCRT machinery to the damaged plasma membrane for shedding through bleb formation, to avoid pyroptosis. While the role of phosphoinositides in the regulation of this process was not evaluated, the potential for some cells to evade death after GSDMD activation may be attributed to phosphoinositide regulation of GSDMD pore and calcium ion dynamics. When taken with reports that phosphoinositides contribute to membrane budding and fusion [162], the ‘bubbling’ morphology that is characteristic of inflammasome-induced pyroptosis could be explained by overwhelming recruitment of the ESCRT machinery to the plasma membrane in an attempt to override cell death, although this requires further study. Other regulators capable of blocking gasdermin dependent pyroptosis have been identified, including apoptotic caspase-3 cleavage and inactivation of GSDMB and GSDMD, and succination of GSDMD by fumarate (an intermediate product of the TCA cycle) [134,163,164], while some evidence exists to suggest that GSDME phosphorylation on tyrosine six blocks its capacity to cause pyroptosis by preventing N-terminal domain oligomerization [165]. The notion that pyroptosis is a passive process with inescapable consequences is antiquated and, therefore, means of its external regulation and potential self-regulation should be explored further.

## Mitochondrial apoptotic BAX and BAK triggering of inflammasome activity and pyroptosis

Mitochondrial apoptosis is executed by BAX and BAK oligomerization and permeabilization of the mitochondrial outer membrane. This allows cytochrome c release, which nucleates the formation of the caspase-9 apoptosome that enables caspase-3 and -7 cleavage and activation, thereby promoting cellular disassembly and clearance in a manner that limits the leakage of immunogenic content [166]. The activity of BAX and BAK is tightly controlled by BCL-2 family members and, in the case of macrophages, pro-survival BCL-2, BCL-xL, MCL-1 and A1 have all been documented as playing important roles in restraining BAX and BAK activation [2,14,167,168].

Despite the assumption that BAX and BAK cause an immunologically silent cell death, recent studies have delineated how BAX and BAK signaling can feed into NLRP3 inflammasome activation and pyroptosis [5,167–172]. Using a cyclic peptolide from myxobacteria, vioprolide A, BCL-2 antagonists (BH3-mimetics) and protein synthesis inhibitors, it was identified that BAX and BAK-triggering result in caspase-3 and -7-mediated potassium ion efflux and NLRP3 activation, in addition to direct caspase-8-induced IL-1β proteolysis [167,168]. Subsequently, it was shown that caspase-3 and -7 activation of the pannexin-1 channel is required for mitochondrial apoptosis-driven NLRP3 signaling [171,172] (Figure 3). The relevance of these *in vitro* genetic and biochemical experiments was highlighted by the discovery that bacterial outer membrane vesicles derived from pathogenic bacteria such as *Neisseria gonorrhoeae* can trigger mitochondrial apoptosis to activate NLRP3 and drive inflammatory IL-1β responses when injected into mice [169]. Similarly, iNOS and caspase-8 can induce a BAX and BAK dependent cell death that is likely to cause damaging immune responses following SARS-CoV-2 infection in mice [14].

Although mitochondrial cell death may cause damaging inflammation in some circumstances, mitochondrial apoptosis is a potent mechanism by which host cells can facilitate clearance of intracellular bacterial and viral infections to prevent lethality in murine models of disease [2,4]. Importantly, mitochondrial apoptotic triggering of pyroptosis is likely conserved in humans as it was recently suggested that, in human skin organoids, mitochondrial-driven caspase-3 and GSDME-mediated pyroptosis blocks the replication of viruses such as herpes simplex virus 1 and vesicular stomatitis virus [5]. Therefore, BH3-mimetics, which have been developed to trigger mitochondrial apoptosis in cancer cells, represent an interesting new approach in the therapeutic treatment of intracellular pathogens, and the above studies indicate that they may cause cell death of infected cells via both mitochondrial apoptotic and pyroptotic signaling

In an interesting twist, research has also uncovered how pore-forming GSDMD and GSMDE can directly target mitochondrial membranes [84,165] (Figure 4). Activated GSDMD and GSDME were reported to interact with and permeabilize mitochondrial membranes to induce the apoptosome downstream of both mitochondrial and death receptor-mediated apoptotic caspase-3 activation, thereby contributing to classical caspase-9 apoptotic signaling [165]. As such, like the feedback-augmentation of apoptotic caspase activity [173], a feedback amplification loop is established whereby GSDME is not only is activated by caspase-3, but also contributes to apoptotic caspase-3 activation via a non-canonical, gasdermin-driven, mitochondrial apoptotic pathway.

## Necroptosis triggering of inflammasome activity

In contrast with pyroptosis and apoptosis, necroptosis is a lytic form of programmed death that is caspase independent and is strictly defined by its requirement for Mixed lineage kinase domain like pseudokinase (MLKL)-mediated membrane damage. Caspase-8 acts as a molecular switch for necroptotic cell death and is essential for inhibiting spontaneous necroptosis in mice [174–176]. As such, necroptosis is thought to have evolved as a host defence against pathogens, specifically those that have developed anti-apoptotic strategies [177]. Following ligand engagement of death receptors or TLRs, caspase-8 complexed with Fas-associated death domain (FADD) can induce extrinsic apoptosis. The TLR adaptor protein TRIF and inhibitor of apoptosis proteins (IAPs; X-linked IAP, cellular IAP1 and IAP2) contribute to this apoptotic death by regulating the ubiquitylation of Receptor interacting protein kinase 1 (RIPK1) and RIPK3 [178,179]. However, upon decreased caspase-8 functioning necroptosis ensues, whereby homotypic interactions between the RIP homotypic interaction motifs (RHIMs) of RIPK1 and the necroptotic effector, RIPK3, initiate RIPK3's phosphorylation of MLKL. Phosphorylation of MLKL drives the oligomerization of its N-terminal and plasma membrane insertion to execute a lytic cell death, being necroptosis [17]. The regulation of necroptosis is tightly controlled at the post-translational level by caspase-8, the deubiquitinases CYLD and A20, and several E3 ubiquitin ligases [180]. Regulation of this lytic death is critical given the release of DAMPs, such as High Mobility Group Box 1 (HMGB1), ATP, IL-33, IL-1α, S100a9 and self-DNA, induce inflammation in surrounding cells [181].

Inflammasome activation downstream of necroptosis is not completely understood and while roles for key necroptotic mediators, RIPK3 and MLKL, have been proposed, further research is required to clarify the different stimuli and cellular contexts that regulate activation, and elucidate its physiological significance. Some of the documented DAMPs released by necroptotic cell lysis have been proposed to induce inflammasome activation [182,183]. However, several studies have also shown that MLKL activity can mediate inflammasome activation downstream of TLR or death receptor signaling [184–186], and that this is caused by potassium ion efflux resulting from MLKL-induced membrane damage, which allows for NLRP3 activation in a cell-intrinsic manner [184] (Figure 3). MLKL triggering of NLRP3 is thereby similar to how GSDMD pores and potassium ion efflux activate NLRP3 following non-canonical inflammasome activation. However, unlike GSDMD being required for efficient IL-1β release following canonical and non-canonical inflammasome signaling, MLKL-driven membrane damage allows the efflux of activated IL-1β from cells upon necroptosis even when GSDMD is deleted [184,185].

The pathological *in vivo* relevance of necroptotic-driven NLRP3 activity is highlighted by the fact that dendritic cell caspase-8 deletion confers susceptibility to endotoxic shock, mediated by RIPK3 and MLKL driven NLRP3-IL-1 activation, but not TNF production [187]. Similarly, A20 deficiency, which causes inflammatory arthritis, results in increases in RIPK1-RIPK3 complexes, RIPK3 and MLKL-mediated NLRP3 inflammasome assembly and unchecked auto-inflammation associated with IL-1β activation [188–192]. Further studies are required to define if IBD and/or arthritis resulting from the loss of other inhibitors of necroptotic cell death, specifically RIPK1 and caspase-8, might drive pathological MLKL-mediated NLRP3 inflammasome responses in humans [193–195].

## Concluding remarks

There is growing evidence supporting the interplay between inflammasome activation and diverse cell death modalities [17,88,196]. Recent work has highlighted the importance of gasdermin family proteins downstream of the inflammasome in mediating pyroptotic cell death, the release of inflammatory cytokines and, by extension, a range of autoinflammatory, infectious and tumorigenic disease states. The potentially interchangeable roles of caspase-1 and caspase-8 in inflammasome signaling offers insight into the complex relationship between pyroptosis and apoptosis, with both pathway intermediates such as caspase-3 and effectors such as GSDMC, GSDMD and GSDME being utilized to steer cells down the various roads to death. Further study of the plasticity of cell death downstream of inflammasome activation, as well as apoptosis and necroptosis, is thus of great importance in understanding the interconnectedness, and potential distinctions, in death signaling, and in informing therapeutic targets of innumerable diseases.

## Abbreviations

AIM2: Absent in melanoma 2
CARD: caspase recruitment domain
DAMPs: damage-associated molecular patterns
ESCRT: endosomal sorting complex required for transport
GAS: group A *Streptococcus*
GPX4: glutathione peroxidase 4
GSDMD: gasdermin D
HMGB1: high mobility group box 1
IBD: inflammatory bowel disease
IFN: interferon
IL: interleukin
LPS: lipopolysaccharide
PAMPs: pathogen-associated molecular patterns
PIP2: phosphatidylinositol 4,5-biphosphate
PJVK: Pejvakin
PLC: phospholipase C
PRRs: pattern recognition receptors
PYD: pyrin domain
RCD1: regulator of cell death 1
RIPK1: receptor interacting protein kinase 1
ROS: reactive oxygen species
TCA: tricarboxylic acid
